# Risk factors for symptomatic Avascular Necrosis (AVN) in a multi-ethnic Systemic Lupus Erythematosus (SLE) cohort

**DOI:** 10.1371/journal.pone.0248845

**Published:** 2021-03-19

**Authors:** Syahrul Sazliyana Shaharir, Siew Huoy Chua, Rozita Mohd, Ruslinda Mustafar, Malehah Mohd Noh, Nor Shuhaila Shahril, Mohd Shahrir Mohamed Said, Sakthiswary Rajalingham

**Affiliations:** 1 Rheumatology Unit, Department of Internal Medicine, Universiti Kebangsaan Malaysia Medical Centre (UKMMC), Kuala Lumpur, Malaysia; 2 Department of Internal Medicine, International Medical University, Kuala Lumpur, Malaysia; 3 Nephrology Unit, Department of Internal Medicine, Universiti Kebangsaan Malaysia Medical Centre (UKMMC), Kuala Lumpur, Malaysia; 4 Department of Medicine, Universiti Malaysia Sabah, Kota Kinabalu, Malaysia; 5 Department of Medicine, Putrajaya Hospital, Putrajaya, Malaysia; Stanford University, UNITED STATES

## Abstract

Avascular necrosis of bone (AVN) is increasingly being recognized as a complication of SLE and causes significant disability due to pain and mobility limitations. We studied the prevalence and factors associated with avascular necrosis (AVN) in a multiethnic SLE cohort. SLE patients who visited the outpatient clinic from October 2017 to April 2019 were considered eligible. Their medical records were reviewed to identify patients who developed symptomatic AVN, as confirmed by either magnetic resonance imaging or plain radiography. Subsequently, their SLE disease characteristics and treatment were compared with the characteristics of patients who did not have AVN. Multivariable logistic regression analyses were performed to determine the independent factors associated with AVN among the multiethnic SLE cohort. A total of 390 patients were recruited, and the majority of them were females (92.6%); the patients were predominantly of Malay ethnicity (59.5%), followed by Chinese (35.9%) and Indian (4.6%). The prevalence of symptomatic AVN was 14.1%, and the mean age of AVN diagnosis was 37.6 ± 14.4 years. Both univariate and multivariable logistic regression analyses revealed that a longer disease duration, high LDL-C (low density lipoprotein cholesterol), positive anti-cardiolipin (aCL) IgG and anti-dsDNA results, a history of an oral prednisolone dose of more than 30 mg daily for at least 4 weeks and osteoporotic fractures were significantly associated with AVN. On the other hand, hydroxychloroquin (HCQ), mycophenolate mofetil (MMF) and bisphosphonate use were associated with a lower risk of AVN. No associations with ethnicity were found. In conclusion, several modifiable risk factors were found to be associated with AVN, and these factors may be used to identify patients who are at high risk of developing such complications. The potential protective effects of HCQ, MMF and bisphosphonates warrant additional studies.

## Introduction

Systemic lupus erythematosus (SLE) is an autoimmune disease that is characterized by multisystem organ inflammation that leads to tissue damage. SLE predominantly affects young women of reproductive age and is associated with a high morbidity rate due to the underlying disease and immunosuppressive treatment. Osteonecrosis or avascular necrosis of the bone (AVN) has been increasingly detected as a complication from SLE since it was first described in 1960 [1]. It usually progresses to irreversible joint damage, causing significant disability due to pain and mobility limitations.

The interruption of blood supply to the bones is the underlying pathogenesis of AVN, and the most commonly affected site is the epiphysis of the femur [2]. Histologically, it is characterized by subchondral bone necrosis as a result of end artery supply to the bone being compromised. The prevalence of symptomatic avascular necrosis (AVN) is reported to be from 0.8%–33%, and that of asymptomatic AVN is reported to be 29–45% among systemic lupus erythematosus (SLE) patients [3]. These large differences in prevalence are due to differences in the study methodology and disease characteristics and sociodemographic backgrounds of the study populations.

Corticosteroids have traditionally been considered the most important risk factor for AVN in SLE [3]; however, AVN has also long been observed in SLE patients who have not been administered corticosteroids [4]. Moreover, high disease activity in the year prior to AVN clinical diagnosis is considered the main independent factor for the development of AVN in SLE [5]. In addition, several other noncorticosteroid-related risk factors for AVN in SLE have been reported, such as arthritis, CNS involvement, diabetes mellitus, hypertension, oral ulcers, renal involvement, vasculitis, a smoking history, leucopenia, thrombocytopenia, cytotoxic drugs and cyclophosphamide [6]. Antiphospholipid syndrome is considered to be one of the risk factors of developing AVN, but conflicting results have been reported in SLE studies.

Avascular necrosis (AVN) is the most frequent condition that occurred in the musculoskeletal system in the local cohort included in our study [7]. US studies have reported a higher prevalence of AVN among African American individuals [8, 9]. Multiethnic studies are scarce, but the Monash Lupus Clinic database demonstrated that there was a trend toward a higher risk of AVN among Asian patients than among non-Asian patients [10]. Therefore, we conducted this study to determine the modifiable risk factors for AVN among a multiethnic Asian SLE population.

## Methodology

### Patients

This was a retrospective study involving patients who visited the UKM Medical Centre (UKMMC) and Putrajaya Hospital from October 2017 to April 2019. All patients fulfilled the Systemic Lupus International Collaborating Clinics (SLICC) classification criteria 2012 [11]. Patients with incomplete medical records and patients who had overlap SLE with other connective tissue disease were excluded from the study. Their medical records were reviewed to identify patients with avascular necrosis (AVN). The diagnosis of AVN was confirmed by imaging, i.e., magnetic resonance imaging (MRI) or radiography. The period from the SLE diagnosis to AVN diagnosis was recorded. Information on the patient’s demographic background (age, sex) and disease characteristics, such as age of onset, disease duration, organ/system involvement, and presence of antiphospholipid antibodies (anti-cardiolipin and lupus anticoagulant), were retrieved from the medical records. The presence of corticosteroid-related complications or damage was recorded, including i) cardiovascular damage, ii) glucocorticoid-induced osteoporosis (GIOP) with fractures, iii) cataracts and iv) corticosteroid-induced diabetes mellitus (CDM).

The medical and prescription records were also reviewed to retrieve the following information on the medication use and treatment of the subjects:

1. cumulative dose of corticosteroids, defined as the sum of the cumulative prednisolone dose and methylprednisolone dose converted to the prednisolone dose.
2. use of more than 30 mg of oral prednisolone daily (>30 mg a day) for at least 4 weeks.
3. use of other immunosuppressants, e.g., azathioprine, mycophenolate mofetil (MMF) and cyclosporine A (CyA), defined as the continuous use of the medications for at least 6 months at the optimum or recommended dose.
4. use of anti-malarial (hydroxychloroquine or chloroquine), defined as the continuous use of a high daily dosage of hydroxychloroquine (i.e., mean daily dosage over the exposure period ≥100 mg for chloroquine and ≥200 mg for hydroxychloroquine) for at least 1 year [12].
5. use of a bisphosphonate agent for prophylaxis of GIOP, as per recommended by the guidelines [13].
6. use of statins to treat dyslipidemia.

For patients with AVN, the above immunosuppressive and treatment information recorded until the date of AVN diagnosis was retrieved. Moreover, for patients without AVN, the information recorded until the last clinic visit was retrieved. Subjects with incomplete treatment records were excluded from the analysis.

This study was approved by the Research and Ethics Committee of the Universiti Kebangsaan Malaysia Medical Center (UKMMC) and Ministry of Health Malaysia (Approval code: FF-2017-425 and NMRR-17-2346-35987). The requirement for informed consent was waived by both ethics committees.

### Statistical analyses

The quantitative variables are reported as the mean and standard deviation (SD) or median and range (minimum-maximum), depending on the normality of the distributions. The normality of the data was assessed with the Kolmogorov-Smirnov test. The absolute and relative frequencies are reported for the categorical variables. AVN patients were evaluated at the time of AVN diagnosis, while other patients were evaluated at the last visit to the clinic during the study period. The incidence rate of AVN was calculated as the number of AVN cases divided by the sum of years of follow-up from the onset of SLE until AVN diagnosis (for AVN cases) or to the last visit to the clinic (for patients without AVN). The SLE clinical and laboratory characteristics were compared between the patients with AVN and without AVN by univariate analysis using χ ^2^ or Fisher’s exact tests (if one or more of the variable values had an expected frequency of five or less) for the categorical variables. In addition, for the continuous variables, independent Student’s t-tests or Mann-Whitney tests were performed, depending on the normality of the data. For comparisons of continuous variables across three or more groups, one-way analysis of variance (ANOVA) was used for normally distributed variables, while the Kruskal-Wallis test was used for nonnormally distributed variables. Multivariable logistic regression analyses were also performed by including all variables that were significant in the univariate analyses, with p<0.05, to determine the independent factors associated with AVN in SLE. Analyses were performed using SPSS, version 21.0 (SPSS Inc. Chicago IL, USA).

## Results

A total of 28 subjects were excluded due to incomplete treatment records, and a total of 390 patients were finally included, with a mean age at the time of study recruitment of 42.8 ± 12.6 years and a disease duration of 16.3 ± 6.8 years. The majority of the patients were females (n = 361, 92.6%), and most were of Malay ethnicity (n = 232, 59.5%), followed by Chinese (n = 140, 35.9%) and Indian (n = 18, 4.6%). More than half of the patients in this cohort had musculoskeletal (65.1%), renal (63.1%) and haematological (60.5%) involvement. A total of 187 patients with LN had renal biopsy. The commonest WHO class of LN was proliferative class III and IV (with or without class V) which affected 76 patients (40.6%) respectively. A total of 22 patients (11.7%) had pure class V LN while 11 (5.88%) had LN class II, and 1 (0.53%) patients each had class I and VI. A total of 11 patients (2.8%) had end stage renal disease (ESRD) and one of them developed AVN 10 years before the onset of ESRD. Seven patients (1.8%) had ischaemic heart disease with one of them had AVN. Three (0.7%) had heart failure and one of them had AVN.

The Chinese patients who were included in this study had longer disease duration, had more LN and anti-dsDNA antibodies than did the Malay and Indian patients (p<0.05). Anti-phospholipid syndrome (APLS) tended to occur more commonly among the Indian patients (p = 0.06). Table 1 illustrates the baseline demographics and disease characteristics of the SLE patients according to their ethnicity.

**Table 1:** Baseline demographics and disease characteristics of the SLE cohort according to ethnicity.

Variables	All (n = 390)	Malay (n = 232, 59.5%)	Chinese (n = 140, 35.9%)	Indian (n = 18, 4.6%)	*p*
Age^a^, mean ± SD (years)	42.8 ± 12.6	41.7 ± 12.5	44.8 ± 12.6	42.1 ± 13.5	0.08
Disease duration^a^, mean ± SD (years)	16.3 ± 6.8	15.4 ± 6.1	17.6 ± 7.7	16.8 ± 6.9	0.01
Age at onset, mean ± SD (years)	29.9 ± 11.9	30.1 ± 11.4	30.2 ± 12.9	26.4 ± 10.4	0.43
Sex					
Female, n (%)	361 (92.6)	211 (91.8)	133 (94.3)	16 (88.9)	0.56
Male, n (%)	29 (7.4)	19 (8.2)	8 (5.7)	2 (11.1)	
System, n (%)					
Musculoskeletal	254 (65.1)	153 (65.9)	86 (61.4)	15 (83.3)	0.17
Renal	246 (63.1)	134 (57.8)	102 (73.6)	9 (50.0)	0.005
Hematological	236 (60.5)	143 (61.6)	83 (59.3)	10 (55.6)	0.82
Mucocutaneous	184 (47.2)	116 (50.0)	58 (41.4)	10 (55.6)	0.21
Neuropsychiatry	51 (13.1)	32 (13.8)	19 (13.6)	0 (0)	0.24
APLS	27 (6.9)	19 (8.2)	5 (3.6)	3 (16.7)	0.06
Lupus nephritis Class					
No biopsy	59 (24.0)	35 (26.1)	23 (22.3)	1 (11.1)	0.56
Class I	1 (0.4)	0	1 (1.0)	0	
Class II	11 (4.5)	7 (5.2)	4 (3.9)	0	
Class III (+/-V)	76 (30.9)	41 (30.6)	29 (28.2)	6 (66.7)	
Class IV (+/-V)	77 (31.3)	37 (27.6)	38 (36.9)	1 (11.1)	
Class V	21 (8.5)	12 (9.0)	8 (7.8)	1 (11.1)	
Class VI	1 (0.4)	1 (0.7)	0	0	
Autoantibody, n (%)					
Anti-dsDNA,	283 (72.6)	157 (67.7)	114 (81.4)	12 (66.7)	0.01
Anticardiolipin IgG^b^	92 (23.6)	53 (23.2)	34 (24.6)	5 (27.8)	0.89
Anticardiolipin IgM^b^	68 (17.4)	39 (17.1)	27 (19.6)	2 (11.1)	0.63
Lupus anticoagulant^c^	38 (9.7)	24 (11.2)	12 (9.6)	2 (11.1)	0.90

APLS = antiphospholipid syndrome, S. D = standard deviation.

^a^at the time of study recruitment.

^b^results available in 384 patients.

^c^results available in 358 patients.

### Prevalence and factors associated with avascular necrosis (AVN)

The point prevalence of avascular necrosis in this study was 14.1% (n = 55), and the overall incidence rate was 9.77 per 1000 patient-years at the follow-up. There was a gradual decline in the incidence rate of AVN, with the calculated incidence rate from the period of 1990–1999 was 29.3 per 1000 patient-years, followed by 13.49 per 1000 patient-years in 2000–2009 and 8.04 per 1000 patient-years in 2010–2019. Majority of the patients had femoral AVN (n = 52, 94.5%). Two patients (3.6%) had AVN involving both femorals and knees while only 1 patient (1.8%) had AVN of the right humeral head. The mean age at onset of AVN was 37.6 ± 14.4 years. The mean disease duration prior to AVN onset was 7.5 ± 6.5 years, with a median of 5 (0.5–29) years. The majority of the patients were in remission when AVN was diagnosed (n = 40, 72.7%) and were taking a median dose of 10 (0–60) mg of oral prednisolone.

The patients who had AVN were significantly older at the time of recruitment and had a longer disease duration, both p<0.05. A significantly higher prevalence of AVN was found among the patients who tested positive for anti-cardiolipin (aCL) IgG, anti-dsDNA, high LDL-C (Low density lipoprotein cholesterol), the patients who had previously received oral prednisolone doses > 30 mg a day and the patients with a history of osteoporotic fractures (all p≤0.05). On the other hand, the patients who received treatment with hydroxychloroquine, azathioprine and mycophenolate mofetil (MMF) had significantly fewer cases of AVN, all p≤0.05. A significantly lower AVN prevalence was also found in the patients who had received bisphosphonates for prophylaxis against corticosteroid-induced osteoporosis (CIOP), p = 0.01.

There was a possibility of the osteoporotic fracture and the use of bisphosphonate groups being less mutually exclusive. This is because as up to 70.6% (n = 24) of patients with osteoporotic fractures received bisphosphonate treatment while only 41% (n = 146) of patients who have no history of osteoporotic fractures received bisphosphonate as prophylaxis against fracture. However, after excluded patients with osteoporotic fracture, the use of bisphosphonate was still significantly associated with a lower prevalence of AVN. A total of 17 (40.5%) patients with AVN had ever received bisphosphonate therapy while 25 patients (59.5%) of the patients with AVN have not received bisphosphonate treatment, p = 0.012. Table 2 illustrates the factors associated with AVN among the SLE cohort.

**Table 2:** Sociodemographic and disease characteristics associated with avascular necrosis among SLE patients.

Variable	No AVN (n = 335)	AVN (n = 55)	*p*
Age^a^, mean ± SD (years)	42.1 ± 12.3	47.6 ± 13.3	0.002
Age at onset (years)	29.9 ± 11.8	30.6 ± 12.2	0.70
Disease duration^a^, mean ± SD (years)	15.6 ± 11.2	20.4 ± 8.8	<0.001
Sex, n (%)			
Male	25 (7.5)	4 (7.3)	
Female	310 (92.5)	51 (92.7)	1.00
Ethnicity, n (%)			
Malay	202 (60.3)	30 (54.5)	0.51
Chinese	119 (35.5)	21 (38.2)	
Indian	14 (4.2)	4 (7.3)	
System/Organ, n (%)			
Lupus Nephritis	211 (63.0)	35 (63.6)	1.00
Hematological	201 (60.0)	35 (63.6)	0.66
Musculoskeletal	217 (64.8)	37 (67.3)	0.76
Mucocutaneous	156 (46.6)	28 (50.9)	0.56
Neuropsychiatric Lupus	42 (12.5)	9 (16.4)	0.39
Antiphospholipid syndrome	23 (6.9)	4 (7.3)	1.00
Vasculitis	58 (17.3)	13 (23.6)	0.26
Proliferative LN^b^, n (%)	130 (81.8)	23 (85.2)	0.79
aCL IgG positive^c^, n (%)	72 (21.8)	20 (37.0)	0.02
aCL IgM positive^c^, n (%)	56 (17.0)	12 (22.2)	0.34
Lupus anticoagulant^d^, n (%)	33 (10.7)	5 (10.2)	1.00
Anti-dsDNA positive, n (%)	237 (70.7)	46 (83.6)	0.05
Immunosuppressants, n (%)			
Corticosteroid use	328 (97.9)	53 (96.4)	0.37
Cyclophosphamide	163 (48.7)	22 (40.0)	0.25
Mycophenolate Mofetil	152 (45.4)	12 (21.8)	0.001
Azathioprine	219 (65.4)	28 (50.9)	0.05
Ciclosporine A	131 (39.1)	17 (30.9)	0.29
Cumulative corticosteroid, mean ± SD (g)	32.9 ± 26.6	29.7 ± 45.3	0.45
Prednisolone > 30 mg a day ^e^, n (%)	103 (30.7)	35 (63.6)	<0.001
Hydroxychloroquine, n (%)	279 (83.3)	25 (45.5)	<0.001
Bisphosphonate, n (%)	208 (62.1)	26 (47.3)	0.05
Corticosteroid-damage, n (%)			
Cardiovascular event	27 (8.1)	5 (9.1)	0.79
CDM	33 (9.9)	8 (14.5)	0.34
Osteoporosis with fracture	21 (6.3)	13 (23.6)	<0.001
Cataract	26 (7.8)	4 (7.3)	1.00
Hypertension, n (%)	170 (50.7)	29 (52.7)	0.88
High LDL-C, n (%)	60 (17.9)	18 (32.7)	0.02
High Triglyceride, n (%)	78 (23.3)	19 (34.5)	0.09

aCL = anticardiolipin, LDL-C = Low density lipoprotein cholesterol, LN = lupus nephritis, CDM = corticosteroid-induced diabetes mellitus, SD = standard deviation.

^a^at the time of study recruitment.

^b^renal biopsy available in 187 patients.

^c^results available in 384 patients.

^d^results available in 358 patients.

^e^prednisolone > 30 mg a day use for at least 4 weeks.

In the logistic regression analysis, which included all variables that had a p value of ≤0.05 in the univariate analyses, the independent factors associated with AVN were aCL IgG positive, anti-dsDNA positive, dyslipidemia, an osteoporotic fracture and a prednisolone dose >30 mg daily. On the other hand, the use of HCQ, MMF and bisphosphonates were associated with a lower risk of AVN (Table 3). The logistic regression model explained 41.9% (Nagelkerke *R*^*2*^) of the variance in AVN and correctly classified 86.1% of the cases.

**Table 3:** Multivariable logistic regression analysis of the independent factors associated with avascular necrosis in SLE.

Parameters	B Coefficient	OR (95% CI)	*P*
Age	0.01	1.01 (0.98–1.04)	0.53
Disease duration	0.05	1.05 (1.00–1.11)	0.05
Hydroxychloroquine use	-1.39	0.25 (0.11–0.54)	<0.001
Osteoporosis with fracture	1.48	4.41 (1.55–12.56)	0.005
Anti-cardiolipin IgG positive	1.08	2.95 (1.35–6.44)	0.007
Anti-dsDNA positive	1.25	3.49 (1.34–9.09)	0.011
Prednisolone > 30 mg daily	1.34	3.84 (1.81–8.11)	<0.001
Mycophenolate mofetil use	-1.53	0.22 (0.08–0.57)	0.002
Azathioprine use	-0.11	0.91 (0.41–1.98)	0.79
Bisphosphonate use	-1.15	0.32 (0.14–0.74)	0.007
High LDL-C	1.27	3.55 (1.53–8.24)	0.003

CI = confidence interval, OR = odds ratio.

Nagelkerke *R*^2^ values = 0.419.

## Discussion

Symptomatic AVN was detected in 14.1% of the SLE patients in our study, which is consistent with the prevalence (12%) reported in other Asian studies [14, 15]. The reported prevalence of symptomatic AVN varies between 0.8% and 33% [3]. An explanation for this discrepancy could be that there is variability in the AVN diagnostic tools, including MRI, conventional radiography and computed tomography, and there are differences in the study methodology. Our study has demonstrated a gradual decline in the incidence rate of AVN over several decades and a similar finding was also observed in a SLE cohort in Hong Kong [16]. An improvement in overall SLE management and the detection of harmful high-dose corticosteroids may have contributed to this decrease in incidence.

Ethnicity may influence individuals’ risk of developing AVN, as African-American patients, including pediatric SLE [3, 17] and kidney transplant patients [18], are particularly predisposed to developing AVN. Several Asian studies have also reported a high rate of AVN, higher than 20%, in areas including Thailand, China, and Hong Kong [16, 19, 20], while the AVN prevalence has been reported to be less than 10% in Caucasian-predominant cohorts [4, 21–29]. Although AVN complications in SLE may merely reflect more severe underlying diseases that require high doses of corticosteroids, a recent study has revealed a possible genetic influence, suggesting that certain ethnicities may be predisposed to developing such complications. For instance, Apolipoprotein L1 (APOL1) variant alleles, and not corticosteroids, were associated with AVN among African American patients with lupus [30]. Moreover, the presence of Multidrug-resistant transporter-1 (MDR1) 3435 TT genotypes was associated with a lower incidence of AVN among Chinese patients with lupus [31]. Genetic variants in the Apolipoprotein L1 (APOL1) gene are found only in individuals with African ancestry. It is one of six members of the APOL gene family on human chromosome located on chromosome 22q12.3. It has an innate immune function which regulates intracellular death and APOL1 gene is associated with an excess risk of chronic and end stage kidney disease [32], as well as atherosclerotic disease in SLE [33]. Meanwhile, Multidrug-resistant transporter-1 (MDR1) gene is located on human chromosome 7 band q21.1. It encodes P-glycoprotein (P-gp), which acts as an energy-dependent membrane efflux pump for various drugs including steroids. It influences the pharmacokinetics of steroids and their metabolites through the pump regulation in absorption and distribution of the drugs at intracellular and extracellular levels. Subsequently this may lead to individual differences in steroid response and sensitivity [34].

Corticosteroid (CS) use has the most robust association with AVN in SLE. The pathogenesis of CS-associated osteonecrosis is not fully understood, but ischemia is the principal pathogenesis of AVN. Fat embolism, an increased intraosseous pressure due to fat cell hypertrophy, vasoconstriction and a reduction in mesenchymal stem cells with corticosteroid therapy are few of the pathomechanisms of corticosteroid-induced AVN [35]. Instead of the cumulative dose, the mean daily or peak dose has been shown to be strongly associated with the AVN dose in a meta-analysis [36]. Our study results concur with the above finding, as no significant associations with cumulative corticosteroid use were found; few patients develop AVN as early as within the first year of SLE diagnosis. The consumption of more than 30 mg of prednisolone daily for at least 4 weeks increased individuals’ risk of developing AVN by 4 times.

However, in SLE, the disease itself, apart from corticosteroids, predisposes patients to developing AVN complications to a greater extent than do other autoimmune chronic diseases with corticosteroid therapy [37]. This finding suggests that the use of corticosteroids may not be the only risk factor associated with the development of AVN in SLE patients. In agreement with this, our study showed that the presence of anti-cardiolipin IgG was one of the predictors of AVN, which is consistent with the results of a few other studies [38, 39]. However, the majority of other studies showed that SLE patients who tested positive for aCL IgM were particularly predisposed to developing AVN complications [3]. Indeed, AVN is recognized as one of the osteoarticular features in primary APLS [40]; thus, it is important to monitor this complication in SLE patients with antiphospholipid antibodies.

A significant association between corticosteroid-induced osteoporosis (CIOP) and AVN in SLE suggests a shared pathogenesis between these complications. Both complications are well known to be associated with corticosteroids in patients with SLE, and there is a significant association between osteopenia and AVN in kidney transplant patients [41]. However, most importantly, our study findings suggest that the use of bisphosphonates as a prophylactic treatment against CIOP may also be beneficial in preventing AVN among SLE patients. Animal studies have demonstrated that bisphosphonates (alendronate) improves the trabecular bone and microcirculation of the femoral head and prevents corticosteroid-induced femoral head AVN in rats [42]. Currently, there are no data on the protective role of bisphosphonates in humans, but there is a small amount of evidence from noncontrolled studies showing that bisphosphonates may play a role in relieving the symptoms of patients who have developed non-femoral head AVN, delaying disease progression and surgery [43]. Thus, in addition to the findings of these studies, our findings suggest that larger clinical trials should be conducted to determine the effectiveness of bisphosphonates in preventing hip AVN.

Another interesting finding to note is that hydroxychloroquine (HCQ) and mycophenolate mofetil (MMF) may have protective effects against hip AVN in SLE patients. There are conflicting results on the protective effect of HCQ. Few studies have demonstrated lower rates of AVN in HCQ-treated patients [15, 19, 39, 44, 45], but a meta-analysis failed to detect such associations [3]. The use of immunosuppressants such as MMF, azathioprine and methotrexate in SLE patients has not been shown to be associated with a higher risk of AVN, except for cyclophosphamide [3]. In contrast, studies among patients who have undergone a hematopoietic transplant showed that the exposure to MMF and calcineurin inhibitors increased their risk of AVN [46].

Dyslipidemia has been reported to be associated with AVN in SLE [14, 17], as intraosseous fat cell hypertrophy and fat embolism are involved in the pathogenesis of AVN. Our study findings concurred with their findings. Patients with LN, especially those with nephrotic range proteinuria, and patients on corticosteroid therapy are indeed predisposed to lipid derangements. A retrospective study reported that statins may lower the risk of AVN in patients who previously received high-dose corticosteroid therapy [47], but additional larger prospective studies are warranted to confirm the effectiveness of statins in preventing AVN.

Our study has several limitations, including its retrospective nature, which leads to many biases. Only symptomatic AVN was diagnosed in this study, and AVN can be asymptomatic in many patients, especially if it occurs at the knees and ankles, and the incidence is when asymptomatic patients are included [3]. In addition, our study did not examine other possible risk factors, such as vitamin D deficiency [48] and obesity [49]. Nevertheless, we revealed several important potential management approaches for preventing AVN among patients with SLE by identifying the risk factors and several potential protective effects of the common medications used in treating SLE patients.

## Supporting information

S1 Appendix Raw data. (SAV)
